# Impact of inaccessible spaces on community participation of people with mobility limitations in Zambia

**DOI:** 10.4102/ajod.v3i1.33

**Published:** 2014-10-14

**Authors:** Martha Banda-Chalwe, Jennifer C. Nitz, Desleigh de Jonge

**Affiliations:** 1 Division of Physiotherapy, University of Queensland, Australia; 2 Division of Occupational Therapy, University of Queensland, Australia

## Abstract

**Background:**

The study investigated the perspective of people with mobility limitations (PWML) in Zambia, firstly of their accessibility to public buildings and spaces, and secondly of how their capacity to participate in a preferred lifestyle has been affected.

**Objectives:**

Firstly to provide insight into the participation experiences of PWML in the social, cultural, economic, political and civic life areas and the relationship of these with disability in Zambia. Secondly to establish how the Zambian disability context shape the experiences of participation by PWML.

**Method:**

A qualitative design was used to gather data from 75 PWML in five of the nine provinces of Zambia. Focus group discussions and personal interviews were used to examine the accessibility of the built environment and how this impacted on the whole family’s participation experiences. The nominal group technique was utilised to rank inaccessible buildings and facilities which posed barriers to opportunities in life areas and how this interfered with the whole family’s lifestyle.

**Results:**

Inaccessibility of education institutions, workplaces and spaces have contributed to reduced participation with negative implications for personal, family, social and economic aspects of the lives of participants. Government buildings, service buildings, and transportation were universally identified as most important but least accessible.

**Conclusion:**

Zambians with mobility limitations have been disadvantaged in accessing services and facilities provided to the public, depriving them and their dependants of full and equitable life participation because of reduced economic capacity. This study will assist in informing government of the need to improve environmental access to enable equal rights for all citizens.

## Introduction

Accessibility of the built environment is regarded as being pivotal to ensuring equity of participation for people with disabilities and has evolved internationally as a topic for concern over recent decades. The built environment can either facilitate or hinder full participation in mainstream society and is considered fundamental to integration, inclusiveness and equality for all as reflected in the United Nations Convention on the Rights of Persons with Disabilities (UN CRPD) (UN Convention 2006; UN 1993; WHO 2001). Specifically, an inaccessible built environment gives an individual with mobility limitation fewer opportunities to participate in education, training and employment, and, limits their experience of positive life situations (Imrie & Hall 2001d; Resnik & Plow 2009; Wee & Paterson 2009).

Of particular importance in this article is the relationship between the individual’s limited participation in consequent education, training and employment opportunities and economic status due to inaccessible built environments and the long-term effects on the family. There is indisputable evidence in many parts of the world (Barnes 1991a; Barnes 2002; Evcil 2009; Fange, Iwarsson & Persson 2002; Imrie & Hall 2001d), that an inaccessible built environment limits the chances for opportunities for people with mobility limitations (PWML) (Imrie & Kumar 2010), making the family less likely to attain a desired economic status. Despite many studies conducted in other countries, to date information is lacking on how participation is influenced by environmental barriers in a developing country such as Zambia. To understand the influence of environmental barriers on participation by PWML, experiences of people with disability (PWD) themselves need to be explored, as relying on proxies to gain this information leads to incomplete data (Bromley, Matthews & Thomas 2007; Hammel *et al*. 2008; Imrie & Kumar 2010).

Access to the built environment is a right for every citizen (UN Convention 2006), regardless of their physical abilities, as it connects the individual to the environment and affords him or her the freedom to participate in different activities of life with ease (Evcil 2009; Iwarsson & Stahl 2003). The International Classification of Functioning Disability and Health (ICF) (WHO 2001), Article 9 of the UN Convention on the Rights of Persons with Disabilities (CRPD) (UN Convention 2006) and Rule 5 of the Standard Rules on the Equalisation of Opportunities for PWD (UN 1993) all advocate for the inclusion of PWD through an accessible environment. Understanding how the built environment affects the participation of people who use mobility devices such as wheelchairs and crutches is important in understanding why an inaccessible built environment should be made accessible.

In most parts of the world, there is ample evidence that the environment negatively affects the participation of people with disabilities, hence efforts to reduce the barriers for example in education and employment (Riddell, Tinklin & Wilson 2004; Rogan *et al*. 2007). Having a paid job is a powerful catalyst for changing other areas of one’s life, for example in social, political, civic and leisure situations, making employment an anchor of a meaningful participation in life (Rogan *et al*. 2007). Rogan and colleagues argue that a paid job would allow an individual to have financial resources to take care of family needs, pay for transport and membership at a social club or political and civic engagements which require finances. However, in most developing countries, Zambia inclusive, the majority of PWD are unemployable due to lack of education opportunities perpetuated by poverty (Eide & Loeb 2006; Filmer 2005; Mitra, Posarac & Vick 2011; SAFOD 2008). The work of Eide and Loeb (2006) on the living conditions of people with activity limitations in Zambia indicated that there are higher percentages in the lower income categories for households with disabled members than households without a disabled member. Their study also indicated that the earnings of most people with disabilities were from unstable income sources and thus indicative of non-formal employment. For example, in the Western province there were significantly more households with a disabled family member who had no one employed (39%) compared to 32% amongst households without a disabled family member. A country profile on promoting the employability and employment of people with disabilities through effective legislation revealed that most disabled persons are not in employment because of inadequate education and training due to the inaccessible built environment and stigma (ILO 2006). These findings are consistent with the available data from the Central Statistical Office in the 2000 national census, which revealed that 43.2% of the 256 690 people with disabilities (2.7% of the total population) had no education and only 1.3% had attained a high level of education (Central Statistical Office 2000). In addition, the census also revealed that 69.2% of them were in self-employment compared to 15.6% in formal employment, and 14.7% were engaged as unpaid family workers. Unfortunately, in Zambia explicit figures to indicate definite employment rates of PWD in comparison to non-disabled populations are scarce. Thus, for the majority of PWD, poverty continues to affect their capacity to educate their children and support their families, and thus their poverty continues in a vicious cycle (Metts 2004; Mitra *et al*. 2011).

There are no known studies in Zambia that have investigated the perspective of PWML regarding accessibility of public buildings and spaces to determine how their capacity to participate in a preferred lifestyle has been affected. Two studies have attempted to include accessibility in their inquiry although, in both cases, accessibility was not their main focus. One was a qualitative study in Lusaka of 24 women with disabilities who reported considerable physical barriers in accessing safe motherhood and reproductive health (RH) services (Smith *et al*. 2004). The other was a cross-sectional study conducted in the nine provinces of Zambia comparing the living conditions of 2885 households (individuals = 15 210) with a family member with a disability (*n* = 2898 PWD) and 2866 households (individuals = 12 979) without a family member with a disability (Eide &Loeb 2006).

This study reported a mixed picture of access to different services, facilities and institutions. For example, less than 40% of PWD who needed banking and hotel services were actually able to access these services. The sample size in this study was large and comprehended all categories of impairments, including sight and hearing. This could have influenced the results, which showed that places of worship, schools, health care clinics and shops were accessible to the majority of those with a disability. Additionally, the results indicated that 65% of PWD accessed public transport and 68% were able to access workplaces. These results could be misleading because of the heterogeneous nature of the sample. Therefore, identifying the built environments that are important in the day-to-day life experiences of PWML for economic development (on PWML and their families) prioritised in this study could reflect more specific outcomes for this group. Hence, this study focused specifically on experiences of accessibility of public buildings in Zambia by PWML and their perception of how inaccessibility affects their participation in their preferred lifestyle.

To fully understand how accessibility of the built environment affects participation of mobility device users, it is critical to identify which environmental features are important in influencing participation (Banda-Chalwe, Nitz & De Jonge 2012b). If participation is a lived experience, then mobility device users may not fully experience participation if they are not able to access life situations such as education, employment, shopping, church services or medical services that form part of this lived experience. Thus, the phenomenological context of the lived experiences of exclusion from participation due to an inaccessible built environment directs the emphasis to the importance of removal of barriers to participation (Patton 2002; UN 2010a; UN 1994). This conceptual understanding of disability, accessibility and participation is lacking in Zambia, despite the Zambian government’s ratification of the UN CRPD, which includes Article 9 on accessibility (UN Convention 2006). However, positive action by government has been demonstrated by the enactment of the *Persons with Disabilities Act* No. 6 of 2012, replacing the *Persons with Disabilities Act* No. 17 of 1996 and reflecting the aspirations of the UN Convention for Persons with Disabilities. This article provides an insight into the participation experiences of PWML in the social, cultural and political areas of life. The article will also demonstrate how these life areas are affected by access barriers which may ultimately lead to them ceasing to participate in the community.

## Purpose

This Zambian study investigates the perspective of PWML regarding accessibility of public buildings and spaces and determines the importance that they place on accessing these areas followed by how their capacity to participate in a preferred lifestyle has been affected due to this inaccessibility.

## Methods

### Study design

A qualitative approach via focus groups and individual interview swas utilised to collect data based on the participants’ own experiences of accessibility (Denzin & Lincoln 2000).

### Study locations

Data were obtained from participants living in five of the nine provinces of Zambia. A stratified, purposive sampling method was utilised to select the five provinces, based on the statistics of PWD and the geographical locations. Physical impairments recorded the highest percentage (38.8%) compared to other impairments (blind, partially sighted, deaf, hearing impairment, mental illness and intellectual impairment) in the 2000 national census of population. The provinces which recorded the highest percentages of people with physical impairment were selected and these were North-Western (46.6%), Southern (43.5%), Eastern (40.3%), Copperbelt (39.1%). However, as the capital city of Zambia as well as the most populous district, Lusaka city (district) in Lusaka province was selected despite having the lowest percentage of PWD (33.8%). The other four provinces were excluded, based on the low percentage of people with physical impairment, namely Central (38.0%), Luapula (37.8%), Northern (36.9%), and Western (35.9%) (Central Statistical Office 2003b). Each of the selected provinces also had unique geographic and socio-economic characteristics; Ndola is the capital city of the Copperbelt mining province, whilst Solwezi is the major rural mining town in the North-Western province. Livingstone is the tourist capital of the country, whilst Chipata is predominantly a major agricultural rural town in the Eastern province. The selection of five study locations was motivated by the fact that this was the first exploratory investigation on the impact of inaccessibility on the lives of people with mobility limitations.

### Participants

Participants included PWML using a wheelchair or crutches for ambulation, aged between 17 and 55 years. Participants were excluded if they were under 12 years (secondary school entry age) and over 55 years (national retirement age), if they presented with cognitive impairment or were unable to provide informed consent to participate. All participants provided informed consent prior to data collection. Most participants understood English even though it was not their first language. However, interpretation of some content of the information sheet was provided by the research assistant for those who were unable to understand the document.

Confidentiality was ensured throughout the process of data collection, management, analysis and publication. The study sought to obtain 100 participants (20 from each identified province) via registers of associations for PWD and government-funded institutions providing services to people with disabilities, aiming for equal representation of gender, age and location. However, communication and transportation difficulties impacted on recruitment numbers in all locations outside Lusaka, particularly Chipata. Participants could not be reached for confirmation of participation and some expressed lack of transport to travel to the interview venue chosen by the Disabled Peoples Organisation (DPO) in that area. Some participants reported their inability to participate due to problems with mobility devices which were either worn out or not repaired because of lack of resources. Thus, participant numbers were augmented with more participants recruited in Lusaka. The distribution of participants in the five locations was: Lusaka 45, Ndola 10, Livingstone 6, Solwezi 11, and Chipata 3. A list of organisations and institutions where the participants were sourced is provided in Appendix 1. The details of the recruitment approach used are described elsewhere (Banda-Chalwe *et al*. 2012b). It should be noted that only 25 individuals who participated in the earlier part of this study contributed data relating to this report. This accounts for the differences in participant numbers between the reports but it did not change the composition or the representativeness of the cohort. Five provinces out of nine were utilised in this study to identify and establish any differences or confirm similarities in experience of barriers in a rural or urban setting, based on the type of buildings or other issues which could be predominant in the different locations.

### Ethical considerations

Ethics approval was sought and obtained from the Medical Research Ethics Committee of the University of Queensland and the Biomedical Research Ethics Committee of the University of Zambia.

### Data collection procedure

After an initial trial conducted in Lusaka to refine the procedure, data collection was undertaken in all five locations between March 2010 and September 2010 comprising ten focus group discussions and seven personal interviews. Personal interviews were utilised to accommodate participants who were unable to attend focus groups due to work commitments and those who felt uncomfortable expressing their views and discussing personal experiences in the presence of other people, especially of the opposite gender. Thus, some preferred to participate in the same gender focus groups whilst others felt it was an opportunity to learn how the other gender experienced barriers of inaccessibility and how it affected them. The data collection process was conducted by the first author with two research assistants who received one day’s training on how to conduct focus group discussions and the use of the nominal group technique. The focus groups and personal interviewees were given unique identifier codes. All focus group discussions and personal interviews were audio recorded in English. Video and photo cameras were also utilised to capture data.

Prior to commencing, demographic data were gathered, including, age, gender, marital status, location, onset of impairment, nature of impairment, years with disability, education level, work, years in employment, support, mobility device and living arrangements. Being guided by the research question, the following open-ended research questions guided the methodology in this study by asking participants:

1. Is accessibility for participation by PWML really a problem in Zambia?
2. Which public buildings do you as PWML regard as important to be accessed?
3. What are the barriers in the built environment that affect your preferred lifestyle participation?
4. How do the barriers you have identified in the built environment affect your participation and your whole family?
5. What accessibility experiences have affected your participation in life areas such as education, training, employment and family responsibilities?

### Focus groups

The focus groups consisted of not more than 15 participants. Each group was constructed according to age and gender to promote belonging and trust, although some groups preferred to be mixed. The questions were asked in the order above.

### Nominal group technique

The nominal group technique (NGT) was originally developed, applied and tested in the late 1960s in the United States of America (USA) by Van de Ven and Delbecq (Gallagher *et al*. 1993; Sample 1984) to generate ideas which are then discussed and ranked by a group.

The process in the current research comprised six steps. Public buildings and spaces that were perceived as important to the participants’ day-to-day life experiences were identified. These were then ranked by the participants into three groups, universally identified as important (on a day-to-day basis), frequently identified as important, and least often identified as important. During the process, each participant was given an opportunity to explain to others in their own words (Claxton, Ritchie & Zaichkowsky 1980) how that building was inaccessible. To satisfy the second part of this study, participants were also encouraged to explain how the inaccessibility of that particular building affected their lives and the whole family’s participation in the community. The process was repeated in a round-robin fashion, ensuring that all participants had an opportunity to identify all the buildings of importance and exhausted their lists. Discussion was encouraged over the rankings accorded to the various public buildings and spaces to permit individuals’ evaluation and to gain consensus (Gallagher *et al*. 1993).

## Data analysis

Simple descriptive statistics were used to analyse participants’ demographic data. Participants were assigned identification numbers, which were applied to audio recorded data from the interviews and focus group discussions. Firstly, recorded data were transcribed in full by the researcher and checked for accuracy and edited by the second and third authors. These transcriptions were entered into the data management program NVivo 10.0 (QSR International 2011) after each interview to store, organise and retrieve data. Secondly, after all interviews were transcribed, codes were developed from emerging issues which represented content descriptors, categories, concepts and themes. The coding of data was conducted by the researcher and two assistants to identify common themes through triangulation of the findings. The codes were examined in order to identify related concepts and stem codes were formed to create a hierarchical structure of issues that had common themes (Bazeley 2007). Lastly, a constant comparative approach between descriptors, categories and concepts of the phenomena was used to obtain common themes. Comparative analysis between focus groups in each location and between the five research locations was also conducted. Comparison was conducted between each theme to identify similarities and differences in descriptions of categories and concepts (Hammel *et al*. 2008). Transcriptions of participants’ interviews from all the five provinces showed no disagreements when compared.

**FIGURE 1:**
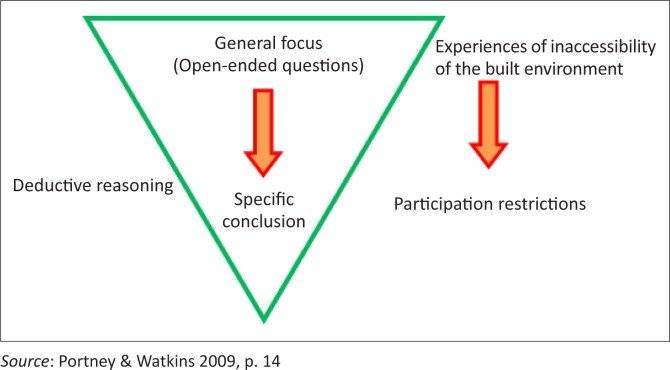
Deductive reasoning in relation to data collection using focus groups.

The concept of deductive reasoning was utilised in data analysis (Figure 1). Deductive reasoning provides a means of understanding, organising phenomena and drawing conclusions from the data itself (Portney & Watkins 2009). In this case, the generalised inaccessibility experience of the built environment by PWML in Western countries might not necessarily represent the experiences of PWML in developing countries (such as Zambia) with different social, political and cultural backgrounds (Marks 1999). Thus, the deductive concept was useful in understanding inaccessibility experiences, organising the data and drawing conclusions on what these experiences mean to PWML in their lives.

The validity of the material reported was ensured through cross examination and comparison of data by the author and assistants to identify common descriptors, categories, concepts and themes between groups and across locations. Validation of data was also conducted by the second and third authors.

## Results

### Study participants

Seventy-five participants were recruited from the five locations in Zambia. The mean age was 36 (SD = 8.7) and 60% were male. The average years with a mobility limitation was 28.3 (SD = 10.5) and a mean of 8.5 (SD = 7.3) years in employment. Wheelchairs were used for mobility by 38.7% and 25.3% lived alone. Only 28% were in full or part time employment, with 50% self-employed (Table 1).

**TABLE 1a:** Demographic characteristics of respondents (*n* = 75).

Description	Sub-category	Frequency (*n* = 75)	%
Gender	Male	45	60
	Female	30	40
Marital status	Married	39	52
	Divorced	8	10.7
	Separated	2	2.7
	Never married	22	29.3
	Widowed	2	2.7
	Not applicable	2	2.7
Location	Lusaka	45	60
	Ndola	10	13.3
	Livingstone	6	8
	Solwezi	11	14.7
	Chipata	3	4
Onset of impairment (self-reported)	At birth	10	13.3
	By 5 years of age	37	49.3
	Between 6 and 11 years of age	12	16
	Adulthood	16	21.3
Nature of impairment (self-reported)	Congenital	10	13.3
	Acquired	65	86.7
	Poliomyelitis	36	48
	Trauma	8	10.7
	Tuberculosis	6	8
	Bewitched	2	2.7
	Unknown origin	13	17.3
Educational level	Primary	12	16
	Secondary	21	28
	Trades/Craft	12	16
	College	17	22.7
	University	9	12
	None	4	5.3
Employment status	Full-time	18	24
	Part-time	3	4
	Retired	2	2.7
	Unemployed – seeking	9	12
	Unemployed – not seeking	1	1.3
	Housewife	0	0
	Self-employed	38	50.7
	Student	4	5.3
Support	Parent	9	12
	Spouse	3	4
	Sister/brother	4	5.3
	Grandparent	0	0
	Self-supported	56	74.7
	Guardian	3	4
Mobility device	Wheelchair	329	38.7
	Crutches	46	61.3
Staying with at home	Alone	19	25.3
	With parent(s)	9	12
	With spouse	24	32
	With family member/relative	23	30.7

**TABLE 1b:** Respondents average age, duration of impairment and employment (*n* = 75).

Description	Mean (Years)	SD (Years)
Age	36	8.7
Duration of impairment	28.3	10.5
Duration of employment	8.5	7.3

SD, standard deviation

### Ranking of public buildings identified as inaccessible

Participants from the 10 focus groups and 7 personal interviews identified public buildings which they needed to access and ranked them as described (Table 2). Even though a home is not a public place, PWML identified it as an important aspect of an individual’s life experiences which also needed to have accessible facilities.

**TABLE 2:** Rank by importance of public buildings identified in day-to-day accessibility experiences of people with mobility limitations (*n* = 75).

Rank order by importance	Name of public building
Rank 1: Universally identified as important (on day-to-day basis)	All government buildings/Ministries
	Public toilets
	Police Stations
	Civic Centres/Municipalities
	Schools
	Colleges
	University of Zambia and other universities
	Hospitals and clinics
	Roads and transportation (public buses, taxis and personal transport)
	Bus stations, bus stops, and train stations
	Markets
	Shopping malls/shops
	Post offices
	Business offices and buildings
	Churches
	Banks
	Homes/houses
Rank 2: Frequently identified as important	Passport offices
	Parliament Building
	Mulungushi International Conference Centre
	Sports and recreation centres
	Airports (International and domestic)
	FINDECO House
Rank 3: Least often identified as important	Zambia Electricity Supply Corporation (ZESCO) offices
	Hotels
	Human Rights Commission
	Social clubs
	Zambia Revenue Authority (ZRA)
	Simoson Building*
	National Building Society (NBS)
	National museums
	Prisons
	Zambia Institute of Special Education (ZAMISE)
	Tourist and leisure resorts

### Experiences of participation restrictions and impact

**FIGURE 2:**
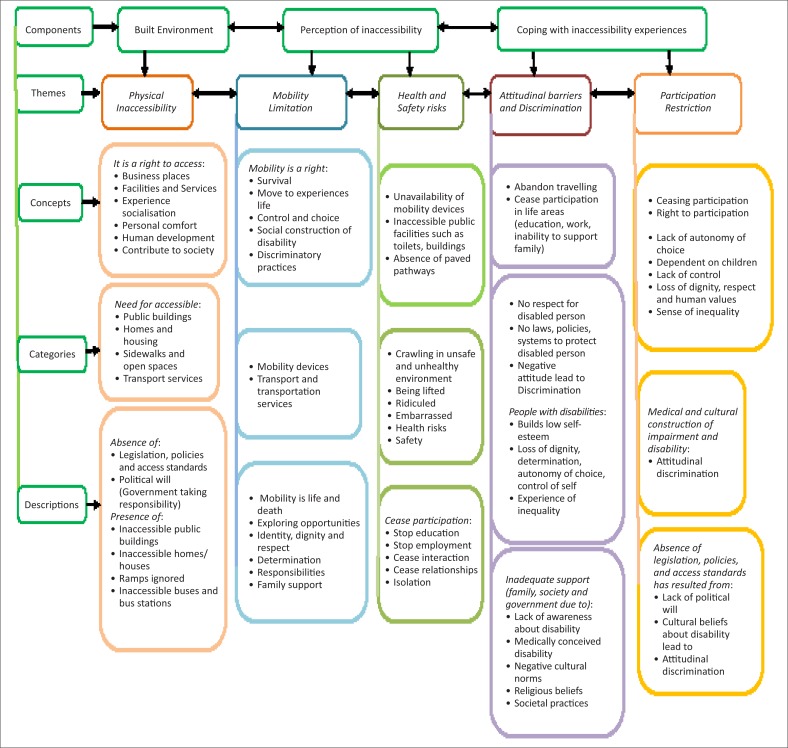
Outcome of focus group responses using the reductive analysis process.

Themes describing participation restrictions and their impact were derived from the recounted experiences of inaccessibility of public buildings and spaces by participants by a process of cross examination and comparison (Figure 2).

Participants identified a number of issues in the built environment, including barriers within society (Figure 2). These are reported under three main sections:

- the built environment, described under
  ▪ physical inaccessibility
- perception of the inaccessibility situation by PWML, described under
  ▪ mobility limitation
  ▪ health and safety risks
- coping with inaccessibility, described under
  ▪ attitudinal barriers and discrimination
  ▪ participation restriction leading to ceasing participation.

However, experiences in each of these different sections interact with each other at various levels, as is evident in the transcribed data contained in this study. Finally, a comparative analysis of the inaccessibility experiences between rural towns and urban cities indicated some differences and similarities.

#### The built environment

**FIGURE 3:**
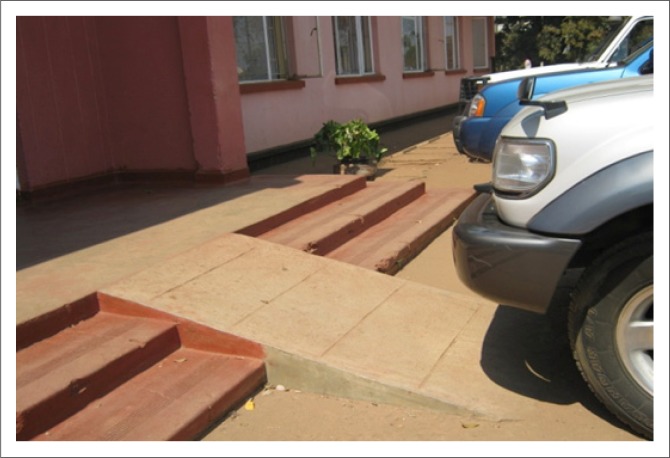
Parked cars obstruct ramp access.

The built environment (public buildings and spaces) was described as a critical aspect of promoting and facilitating participation for PWML. The pursuit of participation opportunities is greatly hindered by barriers encountered in the physical environment (Figure 3 – Figure 6).

**FIGURE 4:**
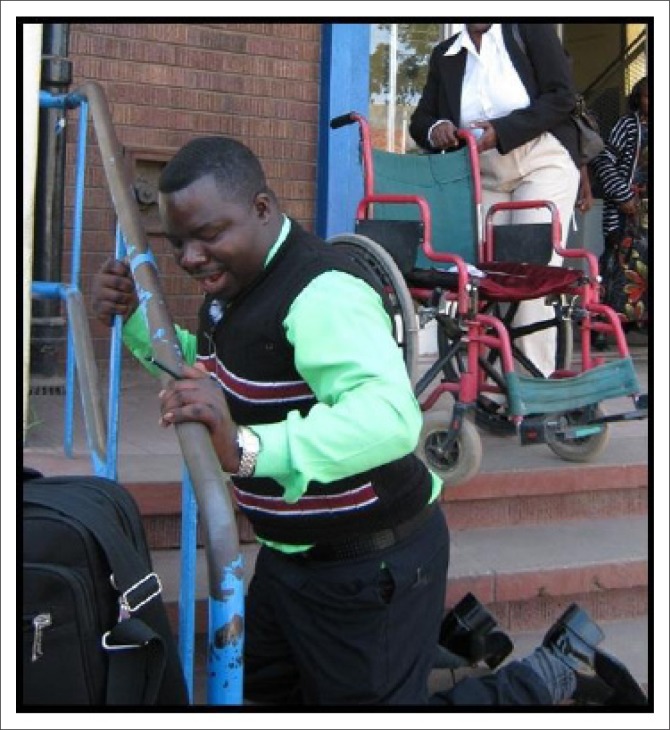
The indignity of crawling up stairs.

**FIGURE 5:**
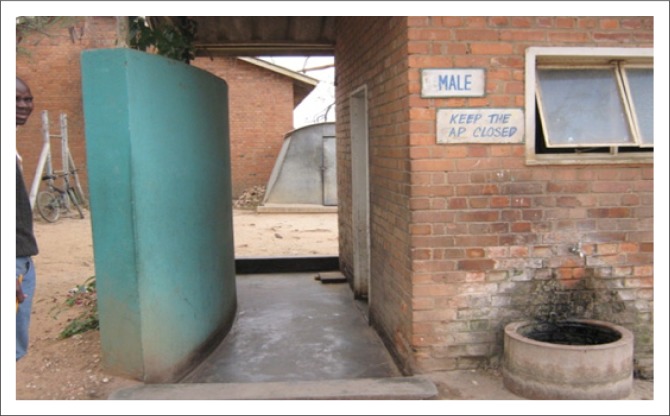
Inaccessible toilet facility.

**FIGURE 6:**
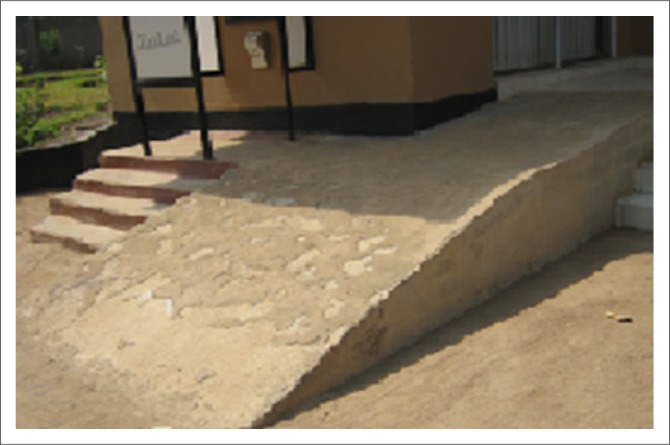
Steep ramp without rails leading to an automated teller machine at a bank.

##### Barriers in the physical environment

Accessibility to the built (physical) environment was described by participants as a right. People with mobility limitations have as much right as other citizens to access business places, facilities and seek services of their choice and experience socialisation. In the process of socialisation, experiences of personal comfort are preferable to struggles in overcoming obstacles and hindrances in the environment. According to the participants, socialisation contributes to an individual’s development, family and to society and this was lacking in their lives.

The need for an accessible built environment – particularly public buildings including homes and houses, sidewalks, open spaces around buildings, roads and parks and transport services – was identified as critical to their participation in life. Comparison of participants’ experiences of barriers encountered in the built environment in urban and rural areas indicated that more buildings in rural areas are at ground level than in urban areas, where many are built with more than one level. Illustrations of these issues have been included in Figure 3 to Figure 6 to allow more understanding of the extent of the problems. Examples from participants describing how they struggle to navigate and wheel their wheelchairs in outdoor areas follow:

‘These obstacles are found everywhere, open drainages along the roads, no designated tarred pathways and high pavements. Mostly I do not go shopping or an outing to a restaurant, even church, I rarely attend.’ (Male aged 46, unemployed but seeking employment)

‘It is too far to wheel myself to and from town on gravel rough pathways and it is also difficult to cross the roads because of high pavements. Too much to think about, so I just stay at home!’ (Male aged 45, self-employed)

‘[*N*]avigating and overcoming obstacles around the school … no paved pathways, uneven surfaces and open drainages I get frustrated and exhausted …’ (Male aged 23, in grade 12)

These statements illustrate that attempts are made to engage in activities such as attending school, visiting business places, shops, leisure and religious facilities, but that the obstacles encountered frustrate the desired efforts. Participants reported different situations which restrict their participation, rendering them unable to fulfil their desired lifestyle. For example, participants wondered why an individual would park a car in front of a ramp, preventing use of the ramp to enable access to a building (Figure 3):

‘You find a car parked in front of a ramp, how do you use it? It means the person who has parked the car has no disability! The use of the International Symbol for Accessibility is not widely known and utilised in our country. Enforcement mechanisms to protect our right to access are also lacking.’ (Male aged 46, full time employed)

Participants viewed blocking an accessibility enabler such as a ramp and disregarding signage indicated disrespect of the person for whom the facility was intended, disregard of the law and reflected ignorance and lack of understanding of the accessibility needs of PWML.

As regards inaccessibility of the physical environment, participants also expressed the general lack of knowledge within the community about the International Symbol for Accessibility and that it was not widely known and used in Zambia. They felt that lack of knowledge about the use of the International Symbol for Accessibility could significantly contribute to the general public not taking recognisance of its use and importance:

‘[*N*]o one seems to be serious about the International Symbol for Accessibility; few people know it exists, what it means and its importance …’ (Male aged 50, self-employed)

Participants blamed government for not committing itself through dissemination of information about disability rights, developing policies and standards and enacting legislation, to initiate the removal of barriers in public buildings and spaces, homes or houses, and transportation services to protect the rights of PWML:

‘I use stairs when I am entering a building because I have no other choice. I crawl up while pulling the wheelchair along or I leave it downstairs and crawl up.’ (Male aged 50, self-employed)

Crawling up and down the stairs of buildings (Figure 4) was identified as an embarrassing experience. As one participant shared his experiences:

‘I crawl up and down stairs of buildings when I want to buy school requirements for my children and the shop where I can find what my children need is upstairs at a shopping complex. I feel embarrassed crawling and my children also feel embarrassed. My wife does not usually accompany me when I am with the children in the city, she feels embarrassed too. What is more critical is the risk that I get exposed to using my bare hands on the floor. Sometimes I am forced to crawl up stairs when I want to attend an interview for a job. Unfortunately, even with all that effort, I do not get the job because I am disabled. Asking me how I would be managing going to the 4th floor crawling if I am offered the job is an insult to my integrity, dignity and respect!’ (Female aged 42, unemployed but seeking employment)

#### Perception of inaccessibility

##### Mobility limitation

Mobility was closely linked by participants to their right to social inclusion. Mobility was expressed as being in pursuit of opportunities for survival, to experience life, and have control of one’s life choices. Participants discussed their experiences of choice and control as lacking in the lives of most PWML in Zambia, as society continues to regard disability as a personal tragedy without relating it to societal discriminatory practices and socio-cultural norms. Mobility was also related to acquisition, use and repair of mobility devices in pursuit of participation and social inclusion. They stated that mobility devices allowed them to move to places of choice and engage in activities of choice, giving them control of what they wanted to do. Without mobility devices, participation was restricted even within the home.

Participants viewed mobility as a matter of life or death. One participant summarised:

‘If you are able to move out and about, you are alive but if you are stationary in your home or bedroom because you cannot move out, then you are as well as dead! The wheelchair provides me with an opportunity to move out and socialise, explore opportunities with dignity and identifying myself as an individual with an impairment and proud the way I am.’ (Female aged 45, unemployed but seeking employment)

Participants expressed determination to take responsibility for their lives and providing family support but that process could only be achieved if they were able to move and not depend on others to do things for them. However, some situations pose a threat to their health and risks are experienced as PWML are determined to pursue participation opportunities.

##### Health and safety risks

Health and safety was discussed regarding health risks, safety and security when pursuing participation activities. Examples related to health risks were outlined as inappropriate architecture (Figure 5), narrow toilet doors, absence of grab rails in toilet rooms and pit latrines in public places. Participants described the necessity to crawl into the toilet area owing to narrow doorways, the small cubicle space, and lack of privacy as they are unable to close the door. Health risk experiences were identified as:

‘Crawling into the toilet room leaving my wheelchair outside is a risk to my health but sometimes I use the inaccessible toilet facility available.’ (Male aged 39, self-employed)

‘“Do you really have to use this dirty toilet?” I am asked a lot of times.’ (Male aged 21, unemployed but seeking employment)

‘I use public toilets without closing the door … the toilet doors and space inside are so small for the wheelchair, no privacy!’ (Female aged 34, full-time employment)

Participants voiced disquiet about how society would expect PWML not to use inaccessible dirty toilets when there is no provision for accessible toilets.

Lack of rails to assist with transfers was seen as additional health risks:

‘Because of having no rails in the toilet room, you have to hold onto the dirty toilet seat to transfer yourself’ (Male aged 43, full-time employment).

Participants also indicated that pit latrines at many schools and churches were barriers to opportunities in these life areas. In addition to crawling into the toilet room, participants described that having to sit on a pit latrine poses a high health risk and may require being lifted by colleagues. Participants described a feeling of embarrassment which may lead PWML to cease pursuing opportunities for participation in areas such as education, employment and religious activities.

Risks were also identified regarding safety issues arising from ramps that were too steep and that lacked rails (Figure 6). Participants described unsafe ramp facilities as creating restrictions for PWML to engage in activities of choice such as using banking facilities:

‘The ramp at the automatic teller machine is so steep, I fail to wheel myself on it and the bank entrance has stairs too. I have closed my bank account! Steep and inappropriate ramps are found not only at bank facilities but even in some schools or churches’. (Male aged 25, training at a college)

Participants related safety risks as contributing to fear of falling, which may result in ceasing interaction and relationships, education and employment. The feeling of insecurity on a steep ramp without rails for support increased the fear of falling.

#### Coping with inaccessibility experiences

Various situations were described as leading to feelings such as anger, frustration, desperation and insecurity. On the other hand, some participants developed motivation and determination to carry on and confront inaccessibility with the assistance of family members such as their children.

##### Attitudinal barriers and discrimination

Attitudinal barriers were common in all five study locations. The attitude of bus conductors and drivers force PWML to abandon travelling. Participants discussed experiences of discrimination based on their impairment and the mobility device they use. The majority of participants indicated that they abandoned school, work, civic and social life events such as cultural ceremonies or sports due to such experiences:

‘It’s how to get to town! Minibus conductors refuse me to get on the bus because of my wheelchair. They say there is no place to put it and I waste time for them. They also charge me for my wheelchair.’ (Female aged 40, unemployed but seeking employment)

‘Minibus drivers leave us at the bus stop/stations because of our wheelchairs and crutches.’ (Female aged 29, unemployed but seeking employment)

In addition, participants felt that society had little or no regard for PWD, reflected in the lack of legislation and policies to protect their rights. They referred to the negative attitudes of society toward PWML leading to discriminatory practices such as denying the right to access opportunities for employment. These discriminatory tendencies could be described by such expressions and comments from participants:

‘When you go looking for a job, mostly you are denied entry to the premises by the security at the gate. In the offices, the negative attitudes of secretaries who will tell you that the interviews are not for persons with disabilities. They really make you feel you are nothing!’ (Female aged 38, unemployed but seeking employment)

‘You cannot perform experiments in the laboratory. It is difficult for you because of your wheelchair.’ (Male aged 21, in grade 12)

‘People don’t see me, they see my wheelchair and judge me because of that!’ (Male aged 34, self-employed)

Participants also experienced people being outspoken about what they should and should not do with their lives, as reflected in the following comments that they recounted from health professionals:

‘“You know that you are disabled, why do you get pregnant?”’ (Female aged 45, unemployed but seeking employment)

‘“Disabled people should not have children, why are you pregnant? You shouldn’t even get married!”’ (Female aged 36, in part-time employment)

**FIGURE 7:**
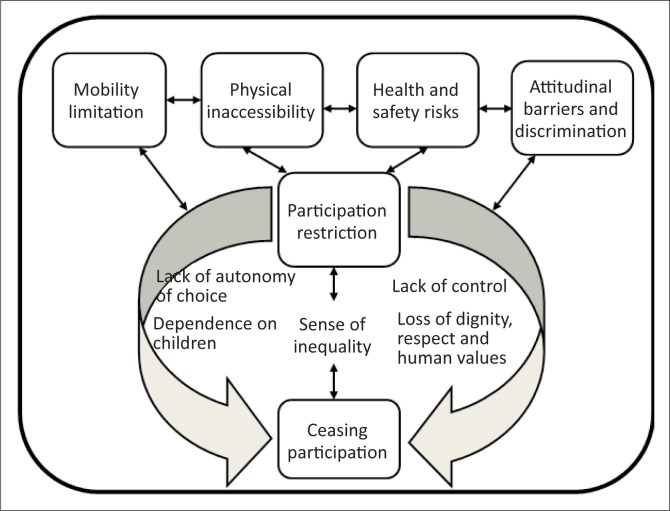
Model of factors that have contributed to ceasing participation by people with mobility limitation.

Attitudinal barriers were related to inadequate support from family, society and government. Participants told how families hide children with disabilities based on traditional beliefs in ancestral curses and cultural practices of consulting traditional healers. A medical-diagnostic perception of disability, for example, by most health professionals was blamed for the belief that a PWML should neither get married nor have children. Health professionals were blamed for the diagnostic and labelling attitude which locates disability within humans and defines it as an anomalous medical condition of long term or permanent duration. Even though some impairment may be long term, participants disliked being labelled and prescribed to by health professionals what they should and should not do due to the impairment. Participants felt that negative comments build in them a sense of low self-esteem, loss of dignity, lack of autonomy of choice, loss of control of one’s life and an experience of inequality (Figure 7). Despite all the negative aspects of coping with inaccessibility, some participants stated that determination to achieve keeps them going:

‘Every day I am lifted up the stairs. It is so embarrassing to be lifted daily … at my work place. There is no ramp leading to where the lift is. I struggle, but I am determined to work’ (Male aged 35, full-time employment).

##### Participation restriction–ceasing participation

Participants emphasised that inaccessibility of the physical environment exposed them to health and safety risks, attitudinal barriers and discrimination, leading to restriction in participation. Experiences of limited mobility were also expressed as leading to ceasing participation as a consequence of inter-related factors such as those identified under perception of inaccessibility (Figure 2). They related participation as a right and experiences of restrictions denied them the right to participate in the lifestyle of their choice. Participation restrictions were also framed as resulting from medical and cultural views of impairment and disability, leading to discriminatory attitudes and practices. The absence of legislation, policies and access standards were identified as a reflection of lack of political recognition on the part of government that perpetuated negative cultural practices from society. Participants emphasised that participation was closely related to the right to social inclusion based on choice and control. In the absence of participation, restrictions pushed PWML into ceasing participation in most areas of life such as education, employment and relationships, leading to isolation, sense of loss and despair, and inability to support their families. At the stage of participation restriction, PWML discussed the sequence of events and experiences prior to ceasing participation. Participants described factors which contribute to ceasing participation, including, (1) lack of autonomy of choice, (2) dependence on others, particularly children, (3) lack of control, (4) loss of dignity, respect and human values and (5) sense of inequality. Ceasing participation was described as an outcome of the other factors listed above. The complex interaction of these observations regarding cessation of participation by PWML reflected experiences common amongst all participants (Figure 7).

##### Lack of autonomy of choice

All participants acknowledged that inaccessibility had created negative attitudes in society which impacted on their autonomy of choice of lifestyle, dignity, privacy and preferences:

‘[*N*]ot much choice of science subjects because laboratories, including the library, are upstairs. No hope of achieving my dreams of becoming an electronic engineer. I know education is my only hope for economic survival …’ (Male aged 18, in grade 11)

‘As for me, society chooses things for me because I cannot access opportunities of my choice on my own … I will always live the dream!’ (Male aged 23, in grade 12)

##### Dependence on children

The use of children as helpers to overcome barriers and obstacles experienced in the built environment was identified as impacting negatively on the education of their children as they spend much of the time meeting their parents’ mobility needs instead of attending school:

‘I always ask my children to push me along on steep slopes or lift me up the stairs and down. My children cannot go to school … The government should make these public buildings accessible so that I can move on our [*sic*] own and my children can also go to school like other children and not be pushing my wheelchair.’ (Male aged 50, unemployed but seeking employment)

##### Lack of control

Little control of one’s own life connected to lack of choice for personal and economic advancement was another critical area identified by participants:

‘I rarely go to the bank. I send my colleagues, sometimes students, to withdraw or bank money for me.’ (Male aged 45, in full-time employment)

##### Loss of dignity, respect and human values

The extent of the impact of negative attitudes of society towards PWD also greatly impacted on the respect, dignity and self-worth and the human values which determine society and how people are viewed and treated:

‘A disabled person has no dignity in life. People do not respect us. They do not think we are human beings.’ (P-Group 1)

‘They look at you as if you don’t need God as they do! Others want to show pity. I don’t like it. I have stopped going to church. Wherever you go it is the same!’ (Male aged 30, unemployed and seeking employment)

##### Sense of inequality

Inequality in life situations related to personal economic advancement further affected their families and wider community, resulting in diminished self-worth due to the inability to contribute economically:

‘Where is equality? Discrimination is seen everywhere! For example, stairs tell you this place is not for you, you don’t belong here!’ (Male aged 44, unemployed but seeking employment)

‘Equality in this country is a dream which I can only imagine when I will be able to go anywhere I want to at any time like everybody else.’ (Male aged 32, unemployed but seeking employment)

‘Society regards us as nothing worth of anything good out of our lives, but more a burden.’ (Group 6)

‘My employers have not provided a ramp for me at the building entrance to access the lift to my office on the fourth floor, yet before I joined the Ministry, a ramp was provided for a senior officer. Despite reporting that I needed a ramp to facilitate access to the location of the lift, the administration has not acted upon my request. I have been here over two years.’ (Male aged 35, full-time employment)

##### Ceasing participation

Ceasing participation altogether was reported as an outcome of the inaccessible built environment, attitudinal barriers and discriminatory tendencies from society:

‘I had to stop engineering because of the inaccessibility of the infrastructure around the university. I could not manage to access the labs, lecture theatres, library and other facilities … there are no ramps or lifts. Also, some comments from people were unpleasant.’ (Male aged 30, in full-time employment)

‘I stopped work because I could not cope being lifted up through the stairs everyday by male security guards. I used to feel embarrassed and humiliated!’ (Female aged 41, self-employed)

‘I don’t go to town most often, because of difficulties in moving on gravel side-road foot paths, up high curbs and corridors along shop buildings, including banks. So I ask my wife to do things for me, even to buy clothes but I would also wish to go myself but …’ (Male aged 40, unemployed but seeking employment)

### Similarities and differences between urban and rural areas

These results reflect common trends across the participants from the five provinces although some differences exist in the types of barriers and how these affected their participation. Similarities in barriers in accessing the physical environment (such as the presence of stairs, inaccessible roads and transportation services) expressed common experiences in all the five locations (rural and urban). Differences were indicated as rural areas having fewer upper storey buildings than urban areas, but more severe transport barriers were reported in rural areas due to rough and uneven terrain on undesignated walkways.

Barriers to transport due to negative attitudes of transporters were reported in all locations, but rural transport costs are higher than urban due to long distances between provincial rural towns and remote villages. Although there are transport services to various locations within cities, people in rural towns walk to most destinations within town unless they use hired taxi, which is unaffordable. In urban cities, transport services are provided using minibuses, while rural towns have few minibuses and instead open vans are used to transport people. Transporters in urban areas are more likely to charge for a wheelchair than in rural areas, even though there are longer distances to travel in rural areas.

Also, there was an indication that the negative attitude towards impairment in rural areas was associated more with cultural views than in urban areas, which showed both cultural and spiritual perspective of disability. Scarcity of cheap, affordable but durable mobility aids is common in all locations. If available, they are donated mobility aids (especially wheelchairs) that are mostly unsuitable for the rough Zambian terrain. Whilst it is difficult to get a wheelchair suitable for individual needs amongst the donated ones, not every PWML even gets a chance to receive a wheelchair. Participants expressed that donated wheelchairs are mostly in urban areas and rarely reach PWML in rural areas except occasionally through the church. Participants in rural areas expressed that the wear and tear of mobility devices was faster due to the rough terrain compared to urban areas which negatively affected their desire to travel.

## Discussion

The purpose of this study was to investigate the perspective of PWML regarding accessibility of public buildings and spaces and to determine how their capacity to participate in a preferred lifestyle had been affected. It was revealed that PWML in Zambia experience accessibility problems related to the buildings as well as transport and public thoroughfares. Inaccessibility affected their ability to make personal lifestyle decisions. Mobility limitation, physical inaccessibility, health and safety risks and attitudinal barriers and discrimination have contributed to participation restrictions for PWML in Zambia. Factors such as lack of autonomy of choice; dependence on children for mobility; lack of control; loss of dignity and respect; and sense of inequality have forced many PWML to cease participation altogether. Inaccessibility of public buildings resulting in limited choices has impacted negatively on the individual economic development and independence of PWML, hence their inability to contribute effectively to the national economy and their families. In addition to inaccessibility experiences, PWML expressed issues which pertain to personal factors, such as personal perceptions and mechanisms to cope with various external factors, which were not originally the focus of this paper. However, the issues highlighted above are pertinent to the way PWML perceive government’s response and involvement in their wellbeing and how they perceive themselves as overcoming these challenges.

Participants identified mostly government buildings (ministries and departments), public institutions such as police stations, post offices and civic buildings and public service providers such as education institutions (schools, colleges and universities), and shopping malls as being public buildings important to their daily life. Despite experiencing mobility limitations, PWML expressed the desire to move out of the confines of home to explore the environment in pursuit of opportunities. Mobility is described as fundamental to the liberty of the human body, and existence and a right to move freely and independently is critical in an individual’s life (Imrie & Hall 2001d). Additionally, in this study, mobility was described as a means of survival and signifies life. Inability to move from one place to another rendered an individual ‘a dead person’.

Movement was aided by the use of mobility devices such as wheelchairs or crutches, which are not readily available in Zambia and, if available, are unaffordable (Handeland, Joelsdottir & Brodtkorb 2008). For example, some potential participants were unable to attend focus group discussions due to having no mobility device for ambulation. Some individuals reported having broken wheelchairs which were beyond repair whilst others were unable to repair their wheelchairs or crutches due to lack of financial resources. Unlike Zimbabwe and South Africa, Zambia has no government-aided wheelchair manufacturing company which can help cushion the cost of a wheelchair. The cost of crutches is equally high for a PWML who is not employed and has no regular income. Crutches are made by community carpenters who charge any desired amount. Other sources are faith-based services such as the Cheshire Homes Society, which mainly provides assistance to children with disabilities within their residential Cheshire Homes through their own sources from the Cross International Catholic Outreach (Cross International 2009). The Cheshire Homes Society receives no funding from government to supply these mobility devices to the general public. The cost of a wheelchair at DISACARE (Disability Care) Wheelchair Centre is about $300. DISACARE, established in 1991 through an initiative of Zambians with disabilities, is the only local NGO aimed at producing durable wheelchairs which are locally built and repairable using locally available raw materials. However, it is not supported by government to cushion the cost (Howard 2003). Most PWD in Zambia are unable to afford the cost of a wheelchair as most of them are unemployed or dependent on small incomes from self-employed activities (Ramsey 2012). The inability to access appropriate and affordable mobility devices also forces PWML to abandon or delay seeking services such as health care (Smith *et al*. 2004), education, training and employment (Lawson 2007; Hurst 1995). Linked to mobility, the cost and scarcity of appropriate mobility devices, and inaccessible public buildings is the inaccessible transportation services previously reported in Zambia (Eide & Loeb 2006; Smith *et al*. 2004) and other countries (Bennett, Lee Kirby & Macdonald 2009; Imrie & Hall 2001d; Metts 2004; Venter, Rickert & Maunder 2003). Despite the government’s acknowledgement of the importance of accessible transportation services, no strategies have been developed to address this need (Ministry of Communications and Transport 2002). In Zambia, transportation services are owned by the private sector, and lack of protective regulations to guide the conduct of transporters renders PWML most vulnerable and subject to abuse by bus drivers and conductors. Participants expressed displeasure at government’s failure to protect their right to access transport, as PWML are being exploited by minibus drivers and conductors who charge for the wheelchair when they are travelling. Deep concern was expressed that the right to mobility was denied to PWML in Zambia due to the inaccessible physical environment and transport.

In addition to inaccessible public transport and transportation services, expressions of despair exist amongst PWML in Zambia regarding the inaccessibility of footpaths and walkways and having to move using their wheelchair and crutches over long distances to various destinations. In rural towns, lack of transport services within the town locations posed more hardships compared to urban cities where minibuses transported people to various locations. In rural towns, people have no option of minibuses, and instead have to walk or travel on wheelchairs to various destinations across the locations or hire a taxi which is unaffordable. As such, the durability of mobility devices was greatly reduced in rural towns compared to urban areas.

Safety concerns were regularly experienced when attempting to access and use public facilities due to obstructions, and poorly constructed elements in the built environment such as high curbs and steep ramps where available. Accessibility issues such as high curbs and a lack of cut-out curbs on road crossings, and rough, uneven and undesignated pathways all posed safety risks. Gravel pathways along roads and within institutional premises such as schools posed major obstacles to accessing services as the provision of paved pedestrian pathways was not part of general construction practice in Zambia. Open drains were also identified as barriers to progression encountered on footpaths. Similar studies conducted in an urban area in Canada (Bennett *et al*. 2009) and a city centre in the UK (Bromley *et al*. 2007) reported the importance of curb-ramps to facilitate easy and safe road crossing.

In Zambia, the absence of ramps or inappropriate ones force PWML to use children as assistants for safety to enable them to overcome obstacles. For example, steep ramps without rails render them functionally inadequate and unsafe and stop PWML visiting places of choice. The practice of using children denies them the opportunity to attend school and poses the risk of illiteracy and future poverty. From a feminist perspective, the rights of both the persons with disabilities and their children are being violated (Morris 2001) in depriving these children of the right to education because of the caring role they assume. By not providing accessible environments, society projects an assumption that PWML are not as important as able bodied people (Imrie & Kumar 2010). The fact that children are providing this care, resulting in the interruption of education and their subsequent earning capacity, has not been hitherto recognised in Zambia. Equally, the consequences of children with mobility impairment leaving school because of inaccessible toilets will perpetuate poverty in adult life for them and their families. Illiteracy has been reported elsewhere as indisputably linked to poverty amongst persons with disabilities (Filmer 2005; Hurst 1995; Lawson 2007; Metts 2004; Trani & Loeb 2010). However, until this study, the link between an inaccessible environment and illiteracy amongst PWML has not been identified in Zambia.

Non-disabled people parking their cars at the end of a ramp, blocking wheelchair access to the building, have been experienced and PWML have been dismayed by this common practice in Zambia. It may be attributed to general lack of knowledge about the needs of PWML by society, lack of accessibility standards and regulations and laws for enforcement. The United Nations has advocated the use of the International Symbol for Accessibility to indicate accessible facilities and services to PWD (UN 2004; UN Convention 2006). This practice has not been promoted and encouraged in Zambia when disseminating information about the needs for PWD.

Barriers within buildings such as narrow doors leading to offices, high reception desks, narrow toilet doors and absence of rails in toilets made facilities in these places inaccessible. High reception facilities in public places such as banks and offices were experienced and this projected a negative perception of society towards wheelchair users (Hurst 1995; Imrie & Kumar 2010), who would be disadvantaged because of height. More importantly, in Zambia, narrow doorways leading to offices or toilet rooms give PWML no option but to crawl into the rooms to use the services. One implication of this is the health risk that the individual is exposed to, which forces PWML to abandon visiting these places altogether. Public knowledge about the need to have accessible toilet facilities was universally expressed as obviously lacking. The lack of knowledge is evident from public reactions when they see an individual with mobility limitations crawling in mostly dirty public toilets. Approaching a solution for better toilet access suitable for PWML through the Ministry of Health under disease prevention might facilitate change since it appears that utilising the perspective of equity of access has not been understood.

Inaccessibility to education and training institutions and employment premises is likely to contribute amongst other factors to the low levels of PWML enrolled in the education system. For example, the living conditions survey reported that disabled children are three times (23.9% of 2885 PWD households with a disabled member) more likely to drop out of school than their non-disabled peers (8.8% of 2866 households without a disabled member) (Eide & Loeb 2006). These results are consistent with the 2004 Ministry of Science, Technology and Vocational Training enrolment in tertiary education system, which recorded only 3% (973/32 841) PWD, comprising 56 who were deaf, 169 with mental impairment, 693 with physical impairment and 55 with visual impairment (MSTVT 2005). The study also indicated that unemployment in Zambia was high and the difference between those with and without disabilities appears to be large, with a significantly higher proportion of people with disabilities (54.5%) not working than amongst people without disabilities (42.0%). These statistics indicate that PWD are disadvantaged from education to employment and hence their living conditions would not be expected to be at the same level as non-disabled individuals.

Inaccessible education and employment environments have been identified elsewhere as contributing to unemployment of PWD (Barnartt 1992; Braithwaite & Mont 2009; Chima 2005; Hurst 1995; Hammel *et al*. 2008; Lawson 2007). Amongst those participants who had managed to gain education, few were employed. Most had been forced into self-employment because inaccessible offices and work environments made formal employment impossible to attain. The inability of an employer or even government ministries to include the provision of an appropriate ramp at the entrance has forced PWML to abandon seeking employment, cease work or resort to self-employment. This situation as well as inaccessible automatic teller machines and banks has interfered with personal control of finances that are necessary components for life participation but denied to PWML in Zambia. The impact due to lack of financial autonomy on participation by PWML has not been reported previously in the literature in Zambia.

Accessibility to premises, facilities and services was described as a right by PWML. Therefore, inaccessibility of the physical environment is a violation of that right. Swain and French (2008) observe that exclusion is the denial of rights and responsibilities of an individual expressed in oppression which shapes the personal and collective experiences and expectations of PWD. Further, it is argued that barriers to participation are the socially constructed oppression through which PWD have to continuously negotiate to gain their rights of access to participation (Swain & French 2008). The UN Convention on the Rights of Persons with Disabilities (UNCRPD) mandates nations to take appropriate measures to identify and eliminate obstacles and barriers to accessibility and ensure that PWD participate fully in all aspects of life (UN Convention 2006) but this has not been embraced by the Zambian government.

According to the UNCRPD, it is government’s responsibility to develop all-inclusive policies and regulations to promote accessible public environments. However, stereotypical and negative attitudes towards PWML pervade government departments. Despite government’s appointment of disability focal point persons (DFPP), negative attitudes have not improved as dissemination of positive information about PWD is lacking as the DFPP have not been trained in disability. Due to their lack of knowledge of disability issues, DFPP have not adequately disseminated information to educate colleagues in government about the needs of PWD, particularly the need for accessibility of the built environment. Professionals such as architects, engineers, lawyers, planners, occupational health officers and physiotherapists (Useh, Moyo & Munyonga 2001), who are viewed as experts on the accessibility needs for all (Church & Marston 2003; Evcil 2009), also have a responsibility to promote accessibility. Architectural design problems were identified here in various areas of the built environment: roads, bus stations, office buildings, churches, sports clubs, shops, banks, educational and health institutions, where the needs of PWML were not met. Architectural considerations in the design of buildings have been identified elsewhere as one critical factor in ensuring accessible public buildings for the participation of PWML within the community (Barnes 1991b; Bromley *et al*. 2007; Hurst 1995; Imrie & Hall 2001c; Imrie & Hall 2001e). Participation was expressed as an outcome of an accessible built environment which benefits the individual, family and society through collective efforts from government, society, professionals and PWD themselves. It was felt that government had not adequately explored the accessibility needs of PWML to promote participation in Zambia.

Stereotypical attitudes of pre-judging an individual with a disability regardless of academic qualifications and skills, the perception by potential employers that a person with a disability could not be productive or attend an interview were prejudices which had contributed to PWML in Zambia not gaining or seeking employment. An example of a participant working for government, denied the provision of a ramp when previously a ramp was provided to a senior government official is a clear indication of government’s failure to take serious action on accessibility. The absence of a system of reporting such violations of disability rights is another failure by government. By allowing a disabled person to be lifted by security guards instead of providing a ramp at the entrance leading to the location of a lift demonstrates how government views PWD. The desire to be seen as human beings and not the disability or the wheelchair or crutches exists amongst PWML in Zambia and, such prejudices may imply that it is the impairment which defines and determines the life chances of an individual (Bromley *et al*. 2007; Morris 2001). To overcome negative attitudes and prejudiced practices from society, most PWML have developed self-determination as the force which has been motivating them to engage in some participation opportunities despite numerous barriers, as has been reported elsewhere (Hammel *et al*. 2008; Gray *et al*. 2008; Wee & Paterson 2009). For example, being told not to get pregnant because of the disability was a clear reflection of how PWML are viewed in Zambia (Smith *et al*. 2004).

The negative attitudes are also reflected in the government’s inability to take a leadership role in identifying the needs of PWML or to institute measures to ensure the provision of an accessible built environment as acknowledged by government (Ministry of Communications and Transport 2002). This situation, if left unchanged, will continue to perpetuate the marginalisation of PWML in Zambia – a sentiment that has been reported elsewhere (Hurst 1995; Lawson 2007; Peat 1997; Venter *et al*. 2003; Wee & Paterson 2009). Going about ones’ own life, doing what one chooses to do, where one wants to go and doing what one wants to do within the environment depends on an accessible built environment (Hammel *et al*. 2006; Imrie & Kumar 2010). This was lacking in experiences of PWML in Zambia.

Much as the attitude of society is critical in initiating change regarding disability concerns, the attitude of PWD toward themselves is equally important. There are several disabled peoples’ organisations (DPOs) in Zambia, but their strength is limited and fragmented due to disunity and limited capacity to lobby government to initiate change (Badley 2008; Handeland *et al*. 2008; ZAFOD 2009). The DPOs in Zambia can learn from similar organisations in other countries – activists such as the Union of the Physically Impaired Against Segregation (UPIAS) and the Liberation Network, and Sisters Against Disability (SAD) in the UK, which were formed to offer powerful mutual support, education and to campaign against discrimination and the oppression of PWD (Barnes & Mercer 2006). In a similar manner, the Disabled Action in New York, USA, was formed in the 1970s, which was also a disability movement with the purpose of engaging in political campaigns and which made a great impact on society towards self-organisation (Barnes & Mercer 2006). From this study, there was an indication that even though PWML in Zambia may know about some of their rights, the capacity to lobby government and society to provide protection of those rights remains with a few educated individuals, some of whom are employed by government and find it difficult to speak against their employers. Participants expressed disappointment at some educated colleagues who are in strategic positions in government ministries but have not shown any indication of advancing the plight of the majority of PWD in the country. However, with the evidence from this study, the DPOs in Zambia could learn from the activities of similar organisations in other countries as disability rights advocacy groups engaging in vigorous campaigns to initiate change of policies on accessibility.

### Study limitations and future research

A limitation of this study is the size of the sample, which translates to 0.08% out of 104 912 persons with physical impairment recorded in the 2000 Zambia national census (Central Statistical Office 2003a). However, owing to the lack of accurate data regarding PWML, a comparative data consideration of 0.08% in a similar study in the USA that utilised 25 individuals in one location out of a total population of 6.8 million PWML (Stark *et al*. 2007) could justify the sample size, which was supported by the purposive recruitment utilised in this study.

The method of recruitment posed a limitation regarding, for example, more participants being in full-time employment (24.0%) compared to those unemployed but seeking employment (12.0%). This could mean that only those who were able to pay for transport to the interview venue managed to attend the focus groups. These results could also indicate that the individuals who possessed communication devices and were reached by the DPOs during the recruitment process were able to come to the focus groups, and excluded potential participants who had no means of communication.

The commonality of the experiences of all participants in this study, regardless of their location, suggests that PWML are universally disadvantaged in Zambia by inaccessible environments and negative attitudes. This study has highlighted a need to establish the extent to which the inaccessible built environment has affected participation in society by PWML. There is a need to quantify the impact of inaccessibility on their life areas and preferred lifestyle to support the claims reported in this study. Also a comparison between public buildings and spaces in rural and urban areas would be essential to establish any differences in the types of buildings and barriers encountered. Such a study is recommended to strengthen this qualitative evidence of inaccessibility of the public buildings and spaces in Zambia.

In addition, future research could focus on a quantitative representative sample on employment and accessibility situation across Zambia. Similar collaborative studies could be conducted in African countries to compare accessibility situations in those nations and establish the magnitude of the problem, as evidence to advance rigorous advocacy across African governments and to political and economic groupings such as the Common Market for Eastern and Sothern Africa (COMESA), Southern African Development Community (SADC) and Economic Community of West African States (ECOWAS). The impact of accessibility in enhancing the achievement of the Millennium Development Goals could also be investigated to establish the level of contribution of PWD participating or not participating in socio-economic development in various countries.

## Conclusion

This study has shown that PWML experience considerable challenges in pursuing opportunities for participation in Zambia. Some of the reported implications from the inaccessible built environment were anger, desperation, dependency, inadequacy, loss of dignity and respect, lack of control of own life, limited choices and inequality in social and family roles, and responsibility. Whilst some of these experiences by PWD have also been reported by the United Nations (UN 2010b) and other studies (Bromley *et al*. 2007; Chima 2005; Hammel *et al*. 2008; Gray *et al*. 2008; Stark *et al*. 2007; Wee & Paterson 2009), this study highlights the dire situation of PWML in Zambia. The acknowledgement by PWML of the country’s limited resources in meeting all the needs of its citizenry was a positive aspect of their high expectation of the government’s role in including accessibility as part of the national agenda and development. The government’s failure in meeting these expectations was expressed in anger, displeasure and desperation. Government is encouraged to take responsibility in meeting the needs of PWML by providing an accessible built environment and initiating measures to ensure equality for all (UN Convention 2006; UN 2010b; Imrie & Hall 2001b; Lutz & Bowers 2005). Drawing from the experiences of inaccessible public buildings by PWML reported in this study, lessons also could be learnt from other countries regarding strategies to improve accessibility and promote participation (Imrie & Hall 2001a; UN 2010a), and this also calls for considerations in systems planning and development (Banda-Chalwe, Nitz & De Jonge 2012a).

## Appendix 1

A purposive sample of 75 persons with mobility limitations was drawn from various sources of registers of associations for persons with disabilities and government funded institutions dealing with disability such as:

- Zambia Agency for Persons with Disabilities (ZAPD) in Lusaka
- Zambia National Association for Women with Disabilities (ZNADWO) in Lusaka
- Zambia Paralympics Committee in Lusaka
- Zambia National Association for the Physically Handicapped (ZNAPH) Wheelchair Centre, Ndola
- DISCARE Zambia, Lusaka
- Livingstone Network of Persons with Disability Organisation
- People Living with Disabilities Support Group, Chipata
- Holland Disabled Association, Solwezi
- Disability Youth Group, Lusaka.
